# Emergency Presentation of Gastric Ectopic Pancreas

**DOI:** 10.7759/cureus.3565

**Published:** 2018-11-08

**Authors:** Pietro Calcagno, Marco Lotti, Luca Campanati, Salvatore Greco, Rosangela Trezzi, Andrea Assolari, Elisa Vaterlini, Cristina Bertani, Niccolò Allievi, Michela Giulii Capponi

**Affiliations:** 1 Surgery, Papa Giovanni Xxiii Hospital, Bergamo, ITA; 2 Internal Medicine, Papa Giovanni Xxiii Hospital, Bergamo, ITA; 3 Patology, Papa Giovanni Xxiii Hospital, Bergamo, ITA

**Keywords:** ectopic pancreas, epigastric pain, pancreatitis, gastritis, gist, net, eus

## Abstract

Ectopic pancreas is a rare embryological abnormality apparently not in association with others. Stomach and duodenum are the most common organs involved. Symptoms are nonspecific. Patients may complain of dyspepsia, abdominal pain or intestinal obstruction. Malignant evolution of ectopic pancreatic cells has been reported. Diagnosis can be very challenging due to the rarity of the disease and the absence of specific symptoms and radiological findings. We report two cases of young-adult men admitted to the emergency department due to acute upper gastro-intestinal and pancreatic symptoms. In both cases, during upper gastrointestinal endoscopy no mucosal vegetations were found. Endoscopic ultrasonography revealed gastric lesions originating from the muscularis propria, with a pattern suspected but not conclusive for malignancy. Fine needle aspiration was inconclusive in both cases. The patients underwent abdominal computed tomography, that showed gastric masses originating from the antrum and the lesser curvature of the stomach, with enlarged locoregional lymph nodes. According to the patients’ symptoms, family history, radiological and cytological findings, the patients were scheduled for an explorative laparoscopy. In both cases, gastric ectopic pancreas was found. Clinical presentation of ectopic pancreas is heterogeneous and the diagnosis can be challenging, especially in an emergency setting. Endoscopic ultrasonography and fine needle aspiration can be useful for the diagnosis and clinical staging, but they can be unspecific. Diagnostic-therapeutic laparoscopy should be considered in symptomatic patients.

## Introduction

The incidence of ectopic pancreas is 0.6–13%, according to autoptic findings [1]. In 90% of the cases, it is found in the submucosal and muscularis propria of the upper gastrointestinal tract, especially in the stomach and the duodenum [2]. Usually, the disease is asymptomatic and the majority of cases are detected incidentally [3]. In rare cases it can present acutely with abdominal pain, vomiting and/or bowel obstruction or thoracic pain [2,4-6]. A few cases of ectopic pancreatic adenocarcinomas are described in the literature [7,8]. Asymptomatic patients with ectopic pancreas and no signs of malignancy can be monitored and do not require treatment [2]. Endoscopic ultrasonography (EUS) is considered the most appropriate diagnostic tool for submucosal gastric lesions. However, it cannot assess the diagnosis with absolute certainty [3]. Accordingly, in symptomatic patients with inconclusive endoscopic, radiological and pathological findings, surgery can be considered for a definitive diagnosis and, possibly, treatment.

## Case presentation

Case report 1

A 21-year-old man was admitted to the emergency department for repeated episodes of vomiting in the previous 24 hours, reporting recurrent dyspepsia and weight loss. His father died of gastric cancer one year before. Abdominal examination was unremarkable except for a mild epigastric tenderness; no masses were appreciated. His blood tests were normal.

**Figure 1:**
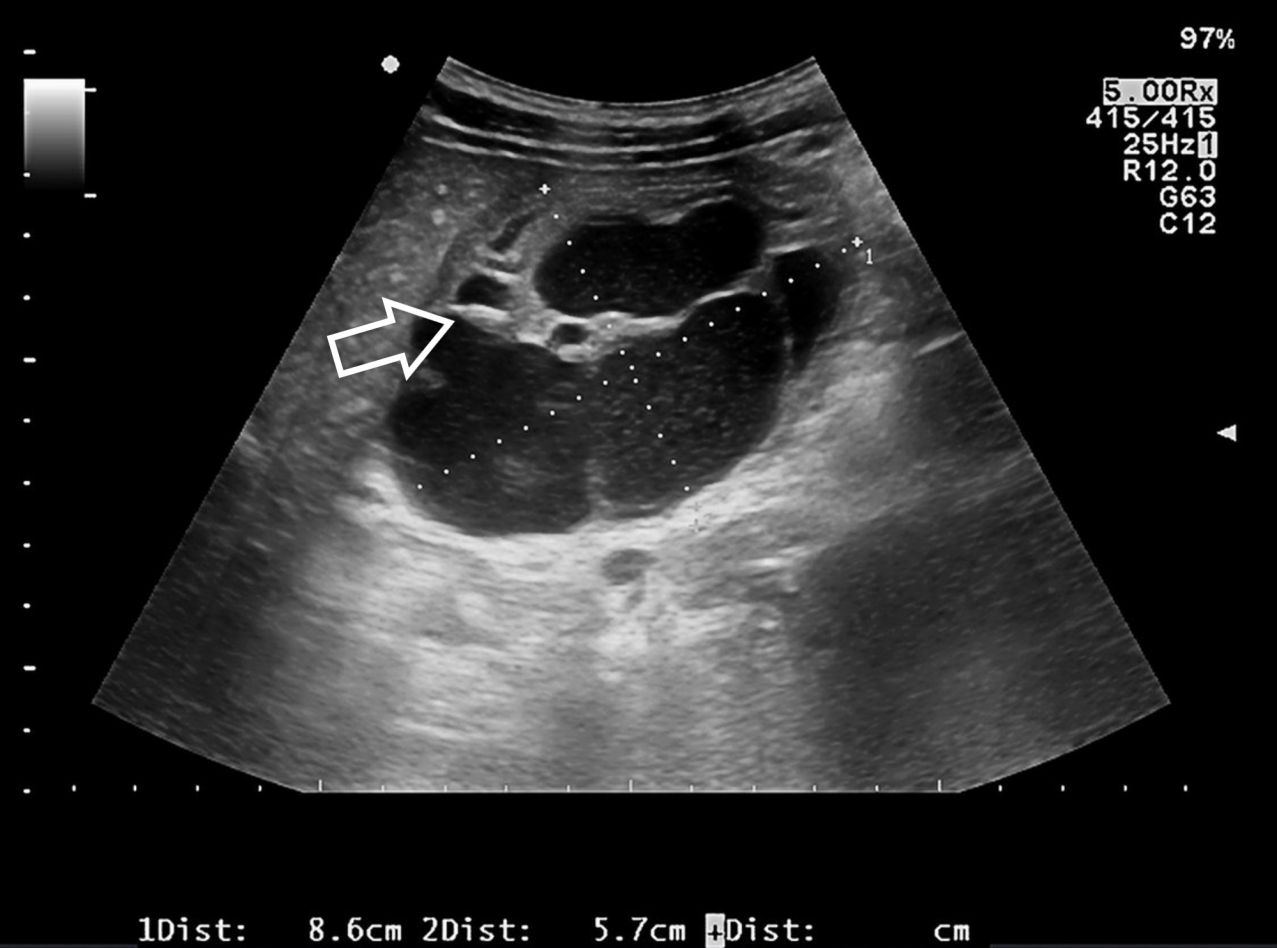
Endoscopic ultrasonography. Heterogeneous mass (8 x 6 cm) in the third and fourth layers of the gastric wall with fluid and a solid (arrow) components.

A plain abdominal radiography showed no free intra-abdominal gas. A nasogastric tube was placed (yielding 150 ml of gastric juice) and the patient was hospitalized. He was treated with starvation, proton pump inhibitors, antiemetics and IV hydration therapy, with good clinical response. According to his personal history, an esophagogastroduodenoscopy (EGD) was performed showing a mucosal bulging without superficial abnormalities. Mucosal biopsies were negative for malignancy. Tumor markers were negative. For a better investigation, the patient underwent EUS (Figure 1): a capsulated, mixed echoic lesion originating from the third and fourth layers of the antral wall, with a nodular hypoechoic portion (diameter 12 mm) and some anechoic components was detected. Fine needle aspiration (FNA) was performed but was inconclusive.

For further investigation, we performed an abdominal computed tomography (CT scan) that showed an 8 x 4 x 5 cm mass originating from the gastric antrum in the lesser curvature, with both fluid and solid well vascularized components and some enlarged celiac and mesocolic lymph nodes.

According to his symptoms, his personal history, the radiological and cytological findings exploratory laparoscopy was indicated. At operation, a huge mass was identified in the posterior wall of the gastric antrum, without serosal invasion. Furthermore, some enlarged celiac lymph nodes were detected and sampled (frozen histology was negative for malignancy). Due to the lesion size and the absence of preoperative histological diagnosis, a laparoscopic distal gastrectomy with D1 lymphadenectomy and Roux-en-Y gastro-jejunal anastomosis were carried out.

Gross histology showed a pancreatic heterotopy without malignancies, localized in the submucosa, muscularis propria and subserosa (Heinrich type I). Eight normal lymph nodes were found.

Case report 2

A 57-year-old man was admitted to the emergency department with acute epigastric and right hypochondriac pain with vomiting; the patient was afebrile. His medical history was positive for obesity, hypertension, recurrent dyspepsia and type II diabetes. No alcohol abuse was declared. At abdominal examination, no masses or tenderness were detected. Blood tests were normal except for white blood cell (WBC) 11000/ml, C-reactive protein (CRP) 4.4 mg/dl, serum lipase 483 U/l. Abdominal ultrasonography showed no gallbladder or biliary tract abnormalities, the pancreatic region was not explorable, no free air was detected. The patient was hospitalized and initially treated with starvation, proton pump inhibitors and IV hydration therapy.

**Figure 2:**
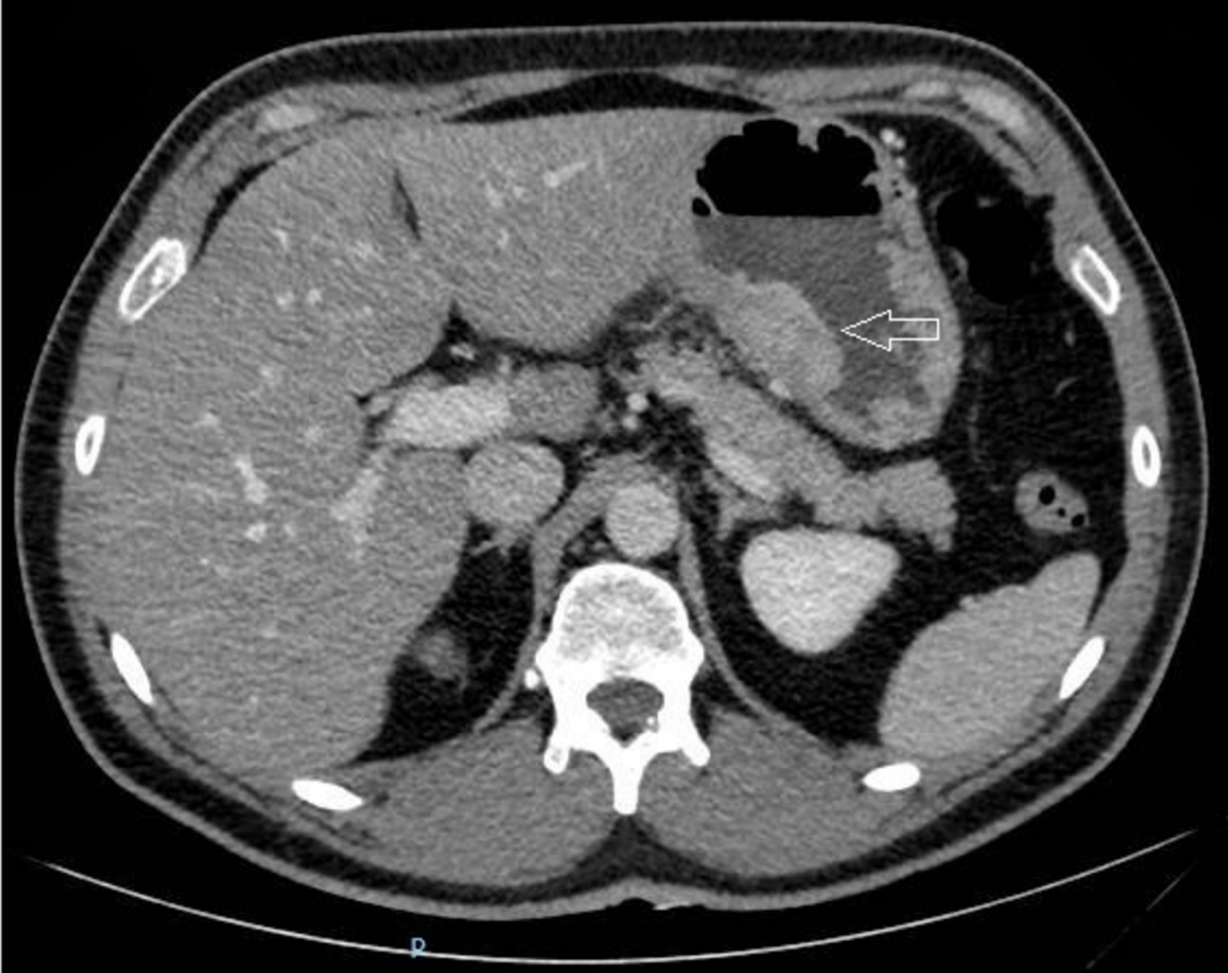
Abdominal computed tomography (CT) scan. Intraparietal nodule (2 x 3 cm) of the gastric lesser curvature (arrow).

In consideration of clinical presentation and unclear first imaging findings, to rule out the suspicion of pancreatitis, an abdominal CT scan was performed 48 hours later (Figure 2): no pancreatic abnormalities were found, while a diffuse gastric wall thickening with a 2 x 3 cm intraparietal nodule in the lesser curvature and some enlarged locoregional lymph nodes were detected.

**Figure 3:**
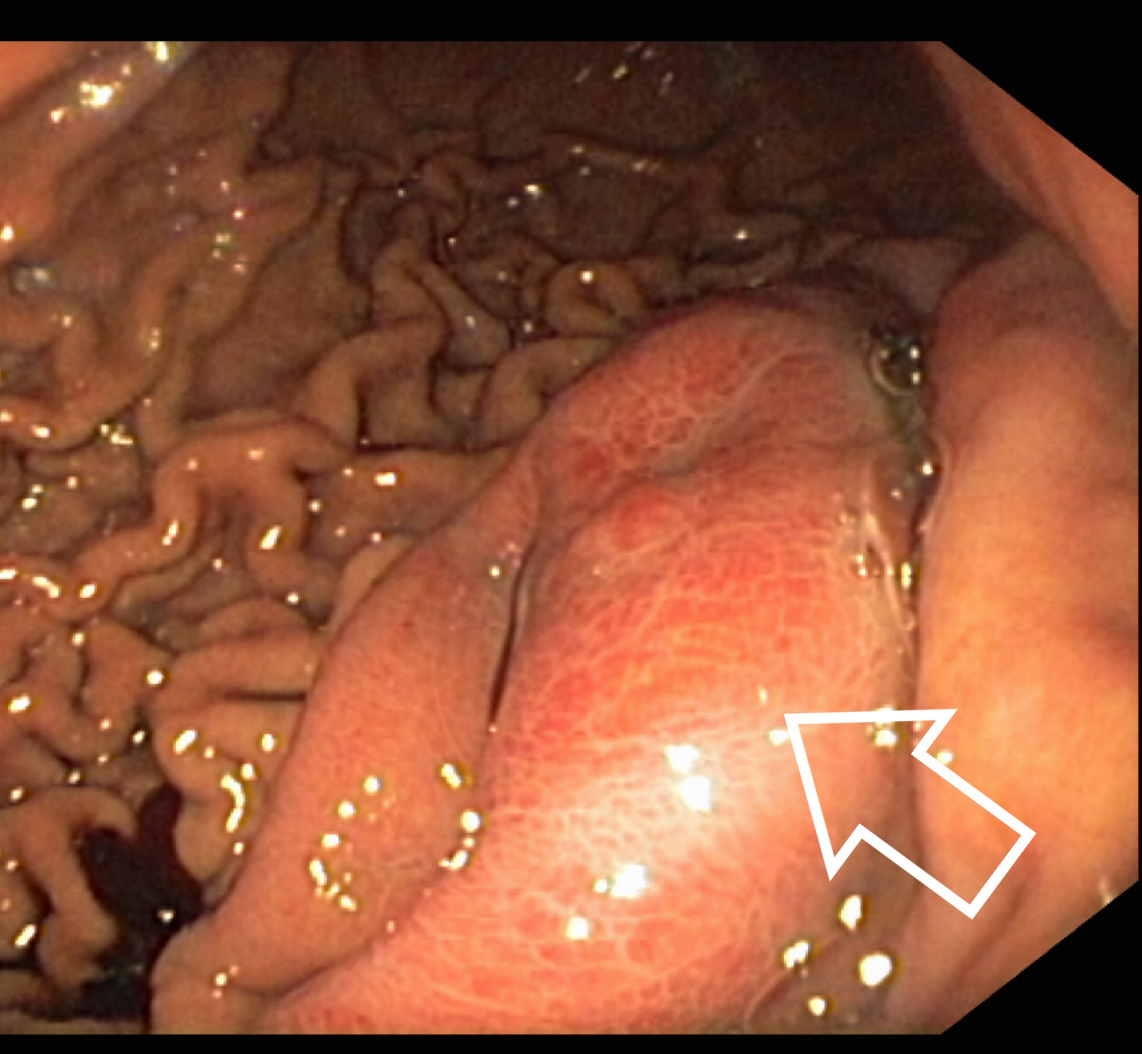
Endoscopic view of the gastric wall. Edematous and hyperemic mucosa without vegetations (arrow).

An EGD (Figure 3) showed marked edema and hyperemia of the mucosa of the gastric body (especially the lesser curvature), but no vegetations were found. Mucosal biopsies were negative for malignancy or gastritis.

**Figure 4:**
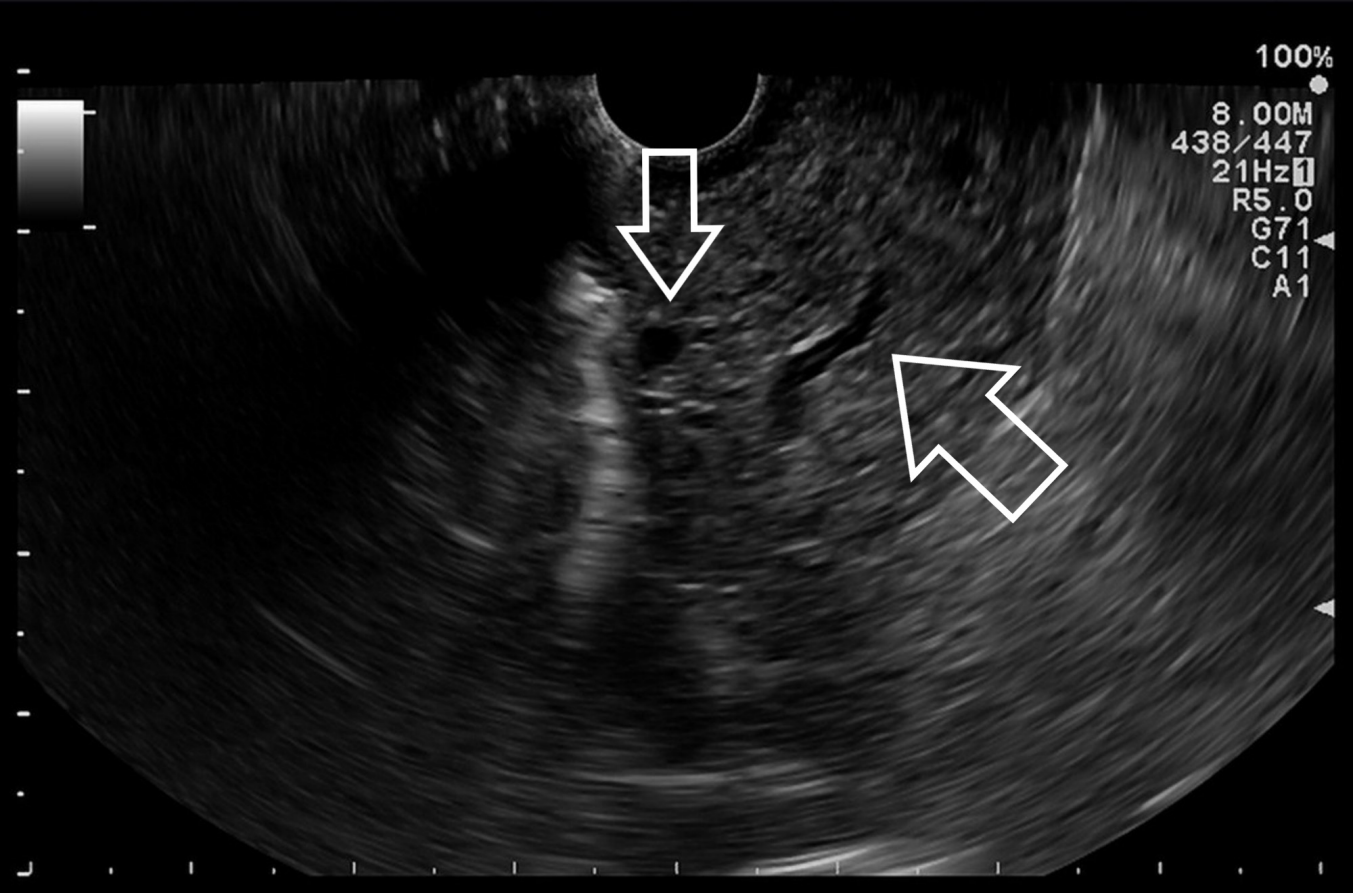
Endoscopic ultrasonography. Mixed echoic mass without a clear distal margin in the fourth layer of the gastric wall; some ductal images are evident (arrows).

At further control, serum lipase value was reduced to 200 U/l and tumor markers were negative. His clinical status gradually improved with medical therapy. After seven days, abdominal CT scan was repeated, showing a reduction in the diameter of his gastric nodule (1.5 x 2.1 cm), while the locoregional lymph nodes were unchanged; no pancreatic abnormalities were detected. For further assessment, EUS was performed (Figure 4): a mixed-hyperechoic lesion with unclear distal margins and some anechoic areas was detected in the fourth layer of the lesser curvature; some enlarged locoregional lymph nodes were found. FNA of the gastric lesion was inconclusive.

Exploratory laparoscopy was finally indicated. Loose adhesions between the posterior wall of the stomach and the pancreatic body were found, especially involving the proximal part of the lesser curvature, where a mass was detected. No serosal invasion was found. Some enlarged celiac lymph nodes were found and sampled (frozen histology was negative for malignancy). An intraoperative EGD was performed to better clarify the proximal margin of the lesion, but it was inconclusive. Therefore, laparotomy was indicated. The lesion proximal margin was identified 3 cm below the cardias and a subtotal gastrectomy with D1 lymphadenectomy and Roux-en-Y gastro-jejunal anastomosis was performed.

**Figure 5:**
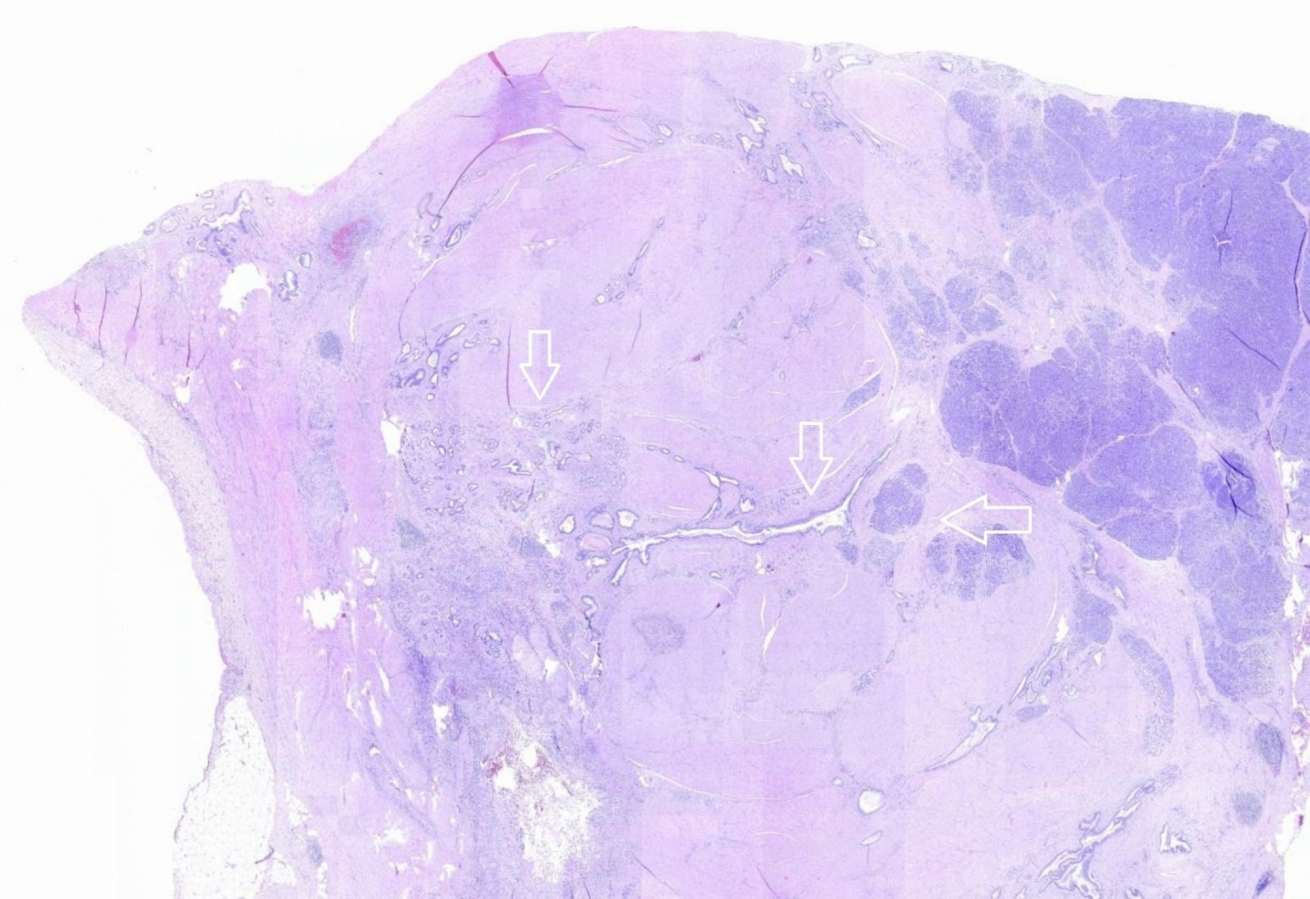
A panoramic view of the gastric wall. Evidence of pancreatic parenchyma regions with ectatic ducts and acini (arrows) in the muscularis propria (Hematoxylin-Eosin, original magnification x10).

**Figure 6:**
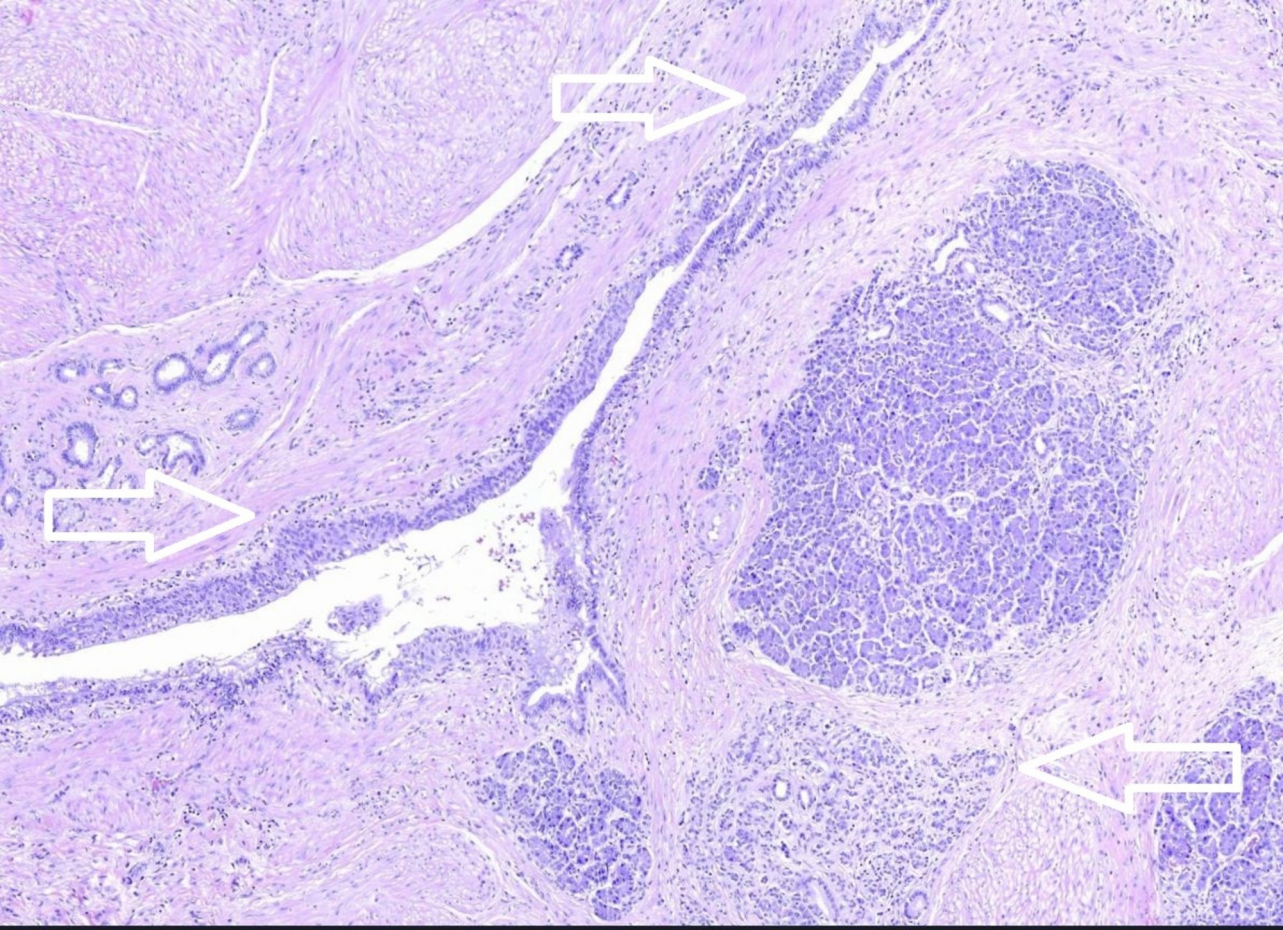
Close up view of the gastric wall. Close up of Figure 5. Evidence of pancreatic parenchyma, ectatic ducts and a diffuse inflammatory reaction (arrows) (Hematoxylin-Eosin, original magnification x10).

At gross histology (Figures 5, 6), ectopic pancreas with signs of chronic inflammation was found in the muscularis propria of the lesser curvature (Heinrich type II). Nineteen normal lymph nodes were identified.

In both cases, the postoperative course was uneventful and the patients were discharged at their seventh postoperative day in good clinical conditions. At clinical follow-up one month later, the patients were asymptomatic and well, and their blood tests were unremarkable.

## Discussion

Pancreatic heterotopia is defined as pancreatic tissue without anatomical or vascular connection to pancreas. It usually occurs in the upper gastrointestinal tract, mainly in the stomach (30%), duodenum (30–90%) and jejunum (20%), the antrum being the commonest affected gastric site [8,9]. Pathogenesis is not clearly understood yet. Pancreatic heterotopia may develop during the embryological foregut rotation, when fragments of pancreas migrate into the upper gastrointestinal tract [7] or can be the result of endodermic metaplasia arising in the submucosal tissue during embryonic life. This second theory explains why ectopic pancreatic cells can be found even in distant anatomical districts like the thoracic cavity [6,7,9].

In a recent retrospective study by Betzler et al. 83.5% of patients with duodenal pancreatic heterotopia were asymptomatic, and diagnosis was made by gross histology after surgery, which was usually performed for biliary or pancreatic neoplasia [8]. In the study by Park et al. 65% of patients with EUS suspicion of gastric ectopic pancreas were asymptomatic [2]. Nevertheless, the disease can be symptomatic. Dyspepsia and epigastric pain are the commonest clinical presentations [2,8]. According to the anatomical site, gastrointestinal obstruction, abdominal pain or thoracic pain may occur [4-6]. Symptoms are mainly related to a mass effect, but in few cases pancreatitis and pseudocysts can develop inside the ectopic pancreas [3,8]: in these cases (like in our second patient) diagnosis can be really challenging.

Malignant degeneration in pancreatic heterotopia is reported [7,8,10] and should be suspected in symptomatic patients. Betzler et al., in their retrospective study, reported two cases (2.9%) of ductal adenocarcinoma arising inside a duodenal ectopic pancreas, both symptomatic [8]. In the previous literature, the prevalence of malignant transformation was 0.7–1.9% [7]. The majority of case reports show that patients with malignant transformation of ectopic pancreas are symptomatic, in case of no mucosal involvement too [3,7].

Diagnosis of the disease is not easy. EGD usually shows only a mucosal bulging. A central dumpling, which corresponds to a duct opening, is present in 35–90% of the cases, but similar findings can occur in case of submucosal gastrointestinal stromal tumors (GIST) or neuroendocrine tumors (NET) [2,3]. Nowadays, EUS is the preferred exam to evaluate submucosal gastric lesions. Ectopic pancreas mean size reported in literature is about 1.3–1.4 cm [2,3]. In a recent review by Kida et al. [9] ectopic pancreas is described as a poorly demarcated, mixed echoic lesion originating from the third layer, sometimes with cystic components. Authors also indicated some EUS signs of malignancy, such as a heterogeneous, nodular component, anechoic areas, origin from fourth layer, ulceration. In these cases, FNA is indicated but its diagnostic yield is approximately 60% [3,9].

Asymptomatic patients usually require monitoring and no treatment. However, Park et al. suggest that endoscopic resection of pancreatic ectopic lesions confined in the submucosa is feasible. They modified a previous EUS classification, creating two groups: S-type lesion (submucosal) and D-type lesion (muscularis propria-subserosal). EUS malignant findings are more common in D-type than S-type [2]. Hence, S-type should be a good candidate for endoscopic removal while D-type require an accurate diagnostic evaluation, and surgery can be considered.

In our first case, the endoscopic exams showed a mucosa bulging. Our second patient did not have bulging but only an edematous, hyperemic mucosa. Both cases did not present any central dumpling and EUS patterns had some malignant stigmata (nodular component, anechoic area, fourth layer involvement). FNA was performed but was inconclusive. Moreover, patients were symptomatic and CT scan showed large size lesions with lymph node enlargement. In our second case, the mass was in the gastric body, where GIST or NET are more common than other submucosal gastric lesions. Therefore, we scheduled the patients for surgery.

## Conclusions

Gastric ectopic pancreas clinical presentation is really heterogeneous and can mimic different pathologies. In this way diagnosis is not easy, especially in an emergency setting. The disease can present itself with malignant degeneration in 0.7–2.9% of cases. EUS with FNA is the preferred exam for clinical staging of submucosal lesions but it cannot assess the diagnosis with absolute certainty. Hence, a diagnostic-therapeutic laparoscopy can be considered in symptomatic patients with large size lesions or findings suspected for malignancy.
